# Effects of B-lymphocyte dysfunction on the serum copper, selenium and zinc levels of rheumatoid arthritis patients

**DOI:** 10.12669/pjms.305.5214

**Published:** 2014

**Authors:** Jiangtao Li, Yan Liang, Hejuan Mao, Wenyu Deng, Jie Zhang

**Affiliations:** 1 Jiangtao Li, Department of Rheumatology and Immunology, West China Hospital, Sichuan University, Chengdu 610041, P. R. China.; 2 Yan Liang, Department of Rheumatology and Immunology, West China Hospital, Sichuan University, Chengdu 610041, P. R. China.; 3 Hejuan Mao, Department of Otolaryngology, The First People’s Hospital of Yibin, Yibin 644000, P. R. China.; 4 Wenyu Deng, Department of Rheumatology and Immunology, The First People’s Hospital of Yibin, Yibin 644000, P. R. China.; 5 Jie Zhang, Department of Rheumatology and Immunology, The First People’s Hospital of Yibin, Yibin 644000, P. R. China.

**Keywords:** B-lymphocyte, Rheumatoid arthritis, Dysfunction, Trace element

## Abstract

***Objective:*** To study the effects of B-lymphocyte dysfunction on the serum copper, selenium and zinc levels of rheumatoid arthritis (RA) patients, and to provide evidence for clinical practice.

***Methods:*** Sixty RA patients enrolled in our hospital from August 2009 to August 2013 were selected as the observation group. Another 60 healthy subjects who received physical examinations in our hospital were selected as the control group. Their B-lymphocyte stimulator (BlyS) levels and CD19^+^CD25^+^ lymphocyte percentages were determined. The levels of trace elements were measured, and correlation analysis was performed.

***Results:*** The BlyS levels of the observation group and the control group were (0.39±0.21) ng/ml and (0.13±0.04) ng/ml respectively, which were significantly different (P<0.05). The percentages of CD25^+^, CD19^+^ and CD19^+^CD25^+^ lymphocytes in the observation group were significantly higher than those in the control group (P<0.05). The serum copper, selenium and zinc levels of the observation group were significantly lower than those of the control group (P<0.05). Pearson's correlation analysis showed that the BlyS level was correlated with the levels of copper, selenium and zinc respectively (r=-0.541, -0.370, -0.430, P<0.05).

***Conclusion:*** Rheumatoid Arthritis may be induced by BlyS-mediated B-lymphocyte dysplasia and dysfunction, accompanied by decreased expressions of copper, selenium and zinc.

## INTRODUCTION

Rheumatoid arthritis (RA) is an autoimmune disease characteristic in synovitis, as well as progressive destructions of cartilage and bone. As one of the main disabling diseases in China, its prevalence rate has reached 1.2%.^1^ Despite of unclear etiology and pathogenesis, RA is generally associated with genetic and environmental factors, and autoimmune response.^2^^,^^3^ B-lymphocyte stimulator (BlyS), which participates in the proliferation and differentiation of B-cells, plays an importance role in humoral immunity.^4^^,^^5^ RA is commonly accompanied by B-lymphocyte dysfunction, which is mainly manifested as evident increase and high activation of CD19^+^CD25^+^ lymphocytes that reflect the disease progress.^6^ It is well known that trace elements are closely associated with many aspects of human life such as tissue construction and repair, immune function enhancement, and even prevention and treatment of RA. As en essential trace element, copper is a crucial component of human proteins and enzymes.^7^ Being accountable for approximately 0.033‰ of body mass, zinc is widely involved in many metabolism processes. Selenium can resist oxidation, boost the immune system, and promote human development and growth, etc.^8^^,^^9^ Therefore, the effects of B-lymphocyte dysfunction on the serum copper, selenium and zinc levels of RA patients were studied herein to provide evidence for clinical practice.

## METHODS

***Subjects: ***Observation group: Sixty RA patients enrolled in our hospital from August 2009 to August 2013 were selected. They were all older than 16, and were diagnosed conforming with the Classification Criteria for Rheumatic Diseases stipulated by American College of Rheumatology in 1987. As first-visit patients, they had not taken slow-acting antirheumatic drugs such as methotrexate and sulfasalazine or biologics. They were not complicated with other autoimmune diseases, cardiovascular and cerebrovascular diseases, metabolic diseases, infectious diseases or liver and kidney diseases. The observation group comprised 32 males and 28 females aged (48.63±3.36) years old in average (youngest: 22; oldest: 69). The average disease course was (20.56±4.12) months (shortest: 6; longest: 35). The average education time was (17.25±4.12) years. Control group: Another 60 healthy subjects who received physical examinations in our hospital were selected. They did not have history of autoimmune diseases, symptoms such as joint pain and swelling, other autoimmune diseases, or hormone treatment. The observation group comprised 31 males and 29 females aged (49.00±3.75) years old in average (youngest: 23; oldest: 70). The average disease course was (20.72±4.00) months (shortest: 5; longest: 36). The average education time was (17.33±4.34) years. The gender, age, disease course and education time of the two groups did not differ significantly (P>0.05). This study was approved by the Ethics Committee of our hospital, and written consent was obtained from all enrolled subjects.

***Determination of B-lymphocytes: ***Blood (3 ml) was collected from the median cubital vein in the morning when the patients were in the fasting state, and was left still for 2 h at room temperature after being treated by anticoagulants. Then the sample was centrifuged at 2500 rpm for 5-10 minutes, and the supernatant was collected and stored at -20°C. Serum BlyS level was measured by ELISA method according to the instructions of the kit provided by Sangon Biotech (Shanghai) Co., Ltd. CD19^+^CD25^+^ lymphocyte percentages were determined by flow cytometry (Epics-XL II, Beckman Coulter, USA) within 6 hour after separating the serum. Relevant antibodies were purchased from Beckman Coulter (USA).

***Determination of trace elements: ***After separating the serum, the levels of trace elements were determined by BH5100S atomic absorption spectrometer (Beijing Bohui Innovation Technology Co., Ltd.) with flame atomic absorption spectrophotometry. All reagents were prepared with deionized water and national standards. Reference ranges: Copper: 11.8-39.3 μmol/L; zinc: 76.5-170.0 μmol/L; selenium: 1.9-2.7 µmol/L.

***Statistical analysis: ***All data were analyzed by SPSS19.0. The categorical data were expressed as (x±s) and compared by independent sample t-test. The numerical data were compared by Chi-square test. Linear correlation analysis was performed to calculate the Pearson’s coefficient. P<0.05 was considered statistically significant.

## RESULTS

***Expressions of serum BlyS: ***The serum BlyS levels of the observation group and the control group were (0.39±0.21) ng/ml and (0.13±0.04) ng/ml respectively, which were significantly different (P<0.05) (Table-I).

**Fig.1:**
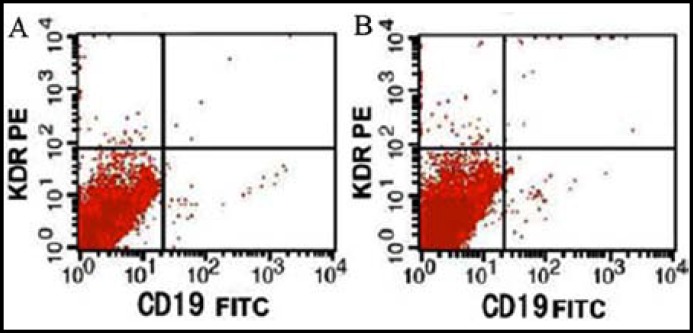
Expressions of serum CD19^+^ cells in peripheral blood. A: Control group; B: observation group

**Fig.2:**
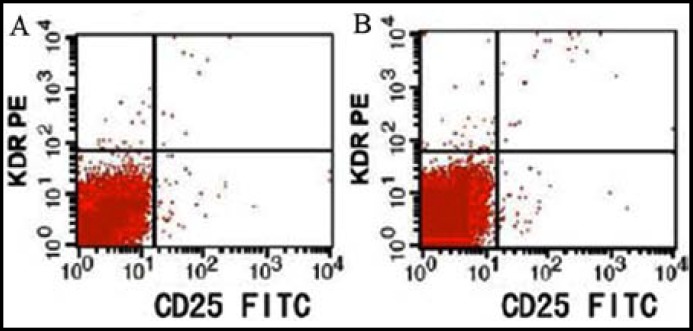
Expressions of serum CD25^+^ cells in peripheral blood. A: Control group; B: observation group.

***Expressions of serum*** ***CD19*** ^+^ ***CD25*** ^+^ ***cells: ***The percentages of CD25^+^, CD19^+^ and CD19^+^CD25^+^ lymphocytes in the observation group significantly exceeded those in the control group (P<0.05) (Table-II, Fig. 1 and Fig. 2).

***Levels of trace elements: ***The serum copper, selenium and zinc levels of the observation group were significantly lower than those of the control group (P<0.05) (Table-III).

***Correlation analysis: ***As evidenced by the Pearson's correlation analysis, the BlyS level was correlated with the levels of copper, selenium and zinc respectively (r=-0.541, -0.370, -0.430, P<0.05) (Table-IV).

## DISCUSSION

As a common, frequently occurring disease, RA endangers human health as an autoimmune disease that is manifested as synovitis and progressive destructions of cartilage and bone in clinical practice. Besides often giving rise to disability, RA also adversely affects the physiology and psychology of patients.^10^ Although ubiquitous among all age groups, RA is more prone to attacking older people. B-lymphocytes play a crucial role in the onset of RA because the involved synovial tissues contain abundant such cells that produce autoantibodies. Meanwhile, B-lymphocytes are antigen-presenting cells for T cells.^11^

**Table-I:** Expressions of serum BlyS (ng/ml, x±s).

***Group***	***Case number (n)***	***BlyS expression level***
Observation group	60	0.39±0.21
Control group	60	0.13±0.04
t		16.521
P		<0.05

**Table-II:** Expressions of serum CD19^+^CD25^+^ cells (%, x±s).

***Group***	***Case number (n)***	***CD25*** ^+^	***CD19*** ^+^	***CD19*** ^+^ ***CD25*** ^+^
Observation group	60	8.81±2.01	10.24±2.11	0.51±0.21
Control group	60	3.23±0.95	6.89±1.05	0.16±0.08
t		13.008	7.248	19.524
P		<0.05	<0.05	<0.05

**Table-III:** Levels of trace elements (μmol/L, x±s).

***Group***	***Case number (n)***	***Copper***	***Zinc***	***Selenium***
Observation group	60	14.36±3.22	86.52±16.36	2.00±0.63
Control group	60	26.86±4.12	141.66±20.00	2.45±0.69
t		9.632	8.412	4.556
P		<0.05	<0.05	<0.05

**Table-IV:** Correlation between B-lymphocyte dysfunction and serum levels of copper, selenium and zinc

***Index***	***Copper***	***Zinc***	***Selenium***
r	-0.541	-0.370	-0.430
P	<0.05	<0.05	<0.05

BlyS is involved in the proliferation and differentiation of B-lymphocytes. In case of over-expression, it is closely related with autoimmune disease, especially RA. In general, BlyS enhances humoral immune response by inducing the proliferation, differentiation and immunoglobulin secretion of B-lymphocytes.^12^ Moreover, BlyS regulates the activation and response of T cells, the over-expression of which may destroy the equilibria of cell production and autoimmune tolerance, thus triggering many types of autoimmune diseases.^13^ In this study, the BlyS levels of the two groups were significantly different, suggesting that the expressions of B-lymphocytes in RA patients were evidently disordered.

For RA patients, B-lymphocytes are the predominant infiltrating cells, and the formation of synovial cell follicles is associated with the level of serum rheumatoid factor.^14^ As a 95 kD type I transmembrane glycoprotein, CD19 is the restricted antigen of B-lymphocytes, so the expression of CD19 corresponds to the level of B-lymphocyte.^15^ Moreover, CD28 molecule is the surface activation marker for B-lymphocytes by being related with their development and maturity. CD19^+^CD25^+^ lymphocytes are the intermediates of B-lymphocyte's differentiation into plasmocyte that can produce antibodies. In this study, the percentages of CD25^+^, CD19^+^ and CD19^+^CD25^+^ lymphocytes in the observation group significantly exceeded those in the control group, indicating that RA patients had abnormally proliferated cell subsets. BlyS may promote the transformation of B-lymphocytes into plasmocytes and boost humoral immune response by increasing the percentage of CD19^+^CD25^+^ lymphocytes.

Of the trace elements in human body, copper is the main component of proteins and enzymes, energizes biochemical processes, contributes to the interaction between collagen and elastin, and affects functions of the immune system.^16^^,^^17^ As the component and activator of some enzymes, zinc is conducive to the growth, development and tissue regeneration of human body, is involved in several immune functions and may protect against the development of RA.^17^^-^19 As the source of life, selenium can resist oxidation, reinforce the immune system, facilitate human development and growth, prevent infectious diseases, and mitigate inflammation induced by autoimmune diseases such as RA and systemic lupus erythematosus.^17^^,^^19^^,^^20^ In this study, the serum levels of copper, selenium and zinc in the observation group were significantly lower than those in the control group. Meanwhile, the BlyS level was correlated with the levels of copper, selenium and zinc respectively (r = -0.541, -0.370, -0.430, P<0.05). Hence, trace elements should be supplemented according to specific symptoms in clinical practice.

In summary, RA may be induced by BlyS-mediated B-lymphocyte dysplasia and dysfunction, which is accompanied by reduced levels of copper, selenium and zinc. Furthermore, the results suggest that BlyS and trace elements may participate in the onset of RA.

## Authors’ contributions:

**LJ and MH:** Design the study and prepared the manuscript;

**LY, DW and ZJ:** Collect and analyze the data.
